# Can baseline ultrasound results help to predict failure to achieve DAS28 remission after 1 year of tight control treatment in early RA patients?

**DOI:** 10.1186/s13075-018-1514-2

**Published:** 2018-01-30

**Authors:** D. F. Ten Cate, J. W. G. Jacobs, W. A. A. Swen, J. M. W. Hazes, M. H. de Jager, N. M. Basoski, C. J. Haagsma, J. J. Luime, A. H. Gerards

**Affiliations:** 1 Department of Rheumatology, Erasmus University Medical Center, Room Na-609; 0031-10-7038251, PO Box 2040, 3000 CA Rotterdam, The Netherlands; 2 Department of Rheumatology and Clinical Immunology, University Medical Center Utrecht, Utrecht, The Netherlands; 3 Rheumatology, Medical Center Alkmaar, Alkmaar, The Netherlands; 4 Rheumatology, Albert Schweitzer Hospital, Dordrecht, The Netherlands; 5 Rheumatology, Maasstad Hospital, Rotterdam, The Netherlands; 6 Rheumatology, Hospital Group Twente, Almelo, The Netherlands; 7 Rheumatology, Vlietland Hospital Schiedam, Schiedam, The Netherlands

**Keywords:** Ultrasonography, Arthritis, Rheumatoid, Prognosis, Remission, Logistic models, Prediction

## Abstract

**Background:**

At present, there are no prognostic parameters unequivocally predicting treatment failure in early rheumatoid arthritis (RA) patients. We investigated whether baseline ultrasonography (US) findings of joints, when added to baseline clinical, laboratory, and radiographical data, could improve prediction of failure to achieve Disease Activity Score assessing 28 joints (DAS28) remission (<2.6) at 1 year in newly diagnosed RA patients.

**Methods:**

A multicentre cohort of newly diagnosed RA patients was followed prospectively for 1 year. US of the hands, wrists, and feet was performed at baseline. Clinical, laboratory, and radiographical parameters were recorded. Primary analysis was the prediction by logistic regression of the absence of DAS28 remission 12 months after diagnosis and start of therapy.

**Results:**

Of 194 patients included, 174 were used for the analysis, with complete data available for 159. In a multivariate model with baseline DAS28 (odds ratio (OR) 1.6, 95% confidence interval (CI) 1.2–2.2), the presence of rheumatoid factor (OR 2.3, 95% CI 1.1–5.1), and type of monitoring strategy (OR 0.2, 95% CI 0.05–0.85), the addition of baseline US results for joints (OR 0.96, 95% CI 0.89–1.04) did not significantly improve the prediction of failure to achieve DAS28 remission (likelihood ratio test, 1.04; *p* = 0.31).

**Conclusion:**

In an early RA population, adding baseline ultrasonography of the hands, wrists, and feet to commonly available baseline characteristics did not improve prediction of failure to achieve DAS28 remission at 12 months.

**Trial registration:**

Clinicaltrials.gov, NCT01752309. Registered on 19 December 2012.

**Electronic supplementary material:**

The online version of this article (10.1186/s13075-018-1514-2) contains supplementary material, which is available to authorized users.

## Background

The prognosis of patients with rheumatoid arthritis (RA) has improved considerably over the past decades, with early intensive treatment with conventional synthetic and biological disease-modifying anti-rheumatic drugs (DMARDs) [1, 2]. Early intensive treatment nowadays is according to ‘treat to target’ [3] and ‘tight control’ paradigms and strategies [4–6]. This has been shown to beneficially alter the long-term outcome of RA patients, especially if applying these strategies early and within the so-called ‘window of opportunity’ [7–11].

Personalised medicine in a ‘treat to target’ and ‘tight control’ setting in RA would require precise and valid instruments predicting which treatment strategy is necessary to achieve the treatment target. These instruments would enable us to decide which patients need more intensive therapy at the initiation of such therapy. Some patients failing to achieve the treatment target of ‘low-disease activity’ (LDA) or remission would have benefitted from combination therapy from the moment of diagnosis, while monotherapy with methotrexate suffices in others. Not many indisputable prognostic parameters in RA patients exist [12]. The parameters most unequivocally predicting a less favourable outcome of RA are high baseline disease activity [13], rheumatoid factor (RF) positivity [14], and peri-articular bone oedema on magnetic resonance imaging (MRI) [15] Arthritis assessed at ultrasonography (US) at baseline might also have predictive abilities since US has been shown to predict progression to RA in undifferentiated arthritis [16, 17]. Furthermore, in RA patients in remission, US results predict the occurrence of flare and erosive progression [18–21]. Based on these results, it has been suggested that we should include joint US findings in remission criteria for RA [22, 23], or to use US for classification criteria of RA [24].

A recent study investigating US predictors for clinical remission did not show US to be predictive for clinical remission. However, in this study there was a more than 50% dropout rate, with significant differences between the tender joint count (TJC) and swollen joint count (SJC) of dropouts and patients included in the final analysis [25]. Thus, in a newly diagnosed RA population starting treatment, it remains equivocal whether baseline US results can help to predict clinical outcomes, as assessed by the Disease Activity Score assessing 28 joints (DAS28) [26–28].

The aim of this study was to see whether a more individualised therapy from the start might be possible in newly diagnosed RA patients who will be treated to target; this is achieved by investigating whether failure to achieve DAS28 remission after 1 year is associated with baseline joint US arthritis findings when added to baseline clinical, laboratory, and radiographical predictors. Secondary aims include associations of joint US arthritis findings with the absence of the EULAR good response criterion after 1 year of therapy and with progression of radiographical joint damage. Additionally, we investigated the association between US joint inflammation at baseline and after 1 year.

## Methods

This was a multicentre (*n* = 7) Dutch study prospectively following for 1 year a cohort of consecutively recruited, newly diagnosed RA patients (1987 ACR criteria) [29], consisting of four sub-cohorts of which two were ongoing clinical trials: the tREACH (Treatment in the Rotterdam Early Arthritis Cohort), with monitoring every 3 months and treatment escalation if the target of low disease activity was not met [30]; and the U-Act-Early trial [31], with monitoring every 4 weeks and treatment escalation as long as remission was not achieved. The third sub-cohort from which patients were included was the “Reumatologie Online Monitoring Applicatie” (ROMA), with monitoring intervals varying from 4 to 12 weeks and treatment escalation according to protocol, as long as remission was not achieved [32]. The fourth sub-cohort consisted of early RA patients from the other centres with monitoring at least every 3 months and treatment escalation according to treat to target, at the discretion of the physician, but following the tREACH protocol (cohort one) as closely as possible. Thus, all patients were treated according to a step-up ‘tight control’ and ‘treat to target’ strategy; treating physicians were blinded to results of US examination. Symptom duration was less than 1 year, and all patients were naive for treatment with DMARDs, including biologicals or glucocorticoids. Treatment strategies included at least a potent conventional synthetic DMARD, such as methotrexate and/or a biological DMARD, with or without glucocorticoids. Prednisolone monotherapy was accepted in nine patients. Exclusion criteria were contraindications for conventional synthetic DMARDs, biological therapy, or glucocorticoids, and illiteracy and personality disorders limiting participation in the study. Patients underwent clinical, laboratory, radiographical, and US joint examination at study enrolment (baseline) and at 1-year follow-up. The study was approved of by the medical ethical committee of the Erasmus MC in Rotterdam. All patients signed a consent form prior to inclusion in the study.

### Clinical, laboratory, and radiographical assessment

Demographics were recorded for each patient. At baseline and at 1 year joint counts included in DAS [33], as well as the patients’ overall assessment of disease activity using a visual analogue scale (VAS), were scored. Functional ability was evaluated using the Dutch Health Assessment Questionnaire (DHAQ) [34] (https://www.nvr.nl/wp-content/uploads/2014/11/NL_consensus_HAQ.pdf), with higher DHAQ scores indicating more disability.

C-reactive protein (CRP) and erythrocyte sedimentation rate (ESR) were obtained at both visits; RF and anti-cyclic citrullinated peptide (aCCP) status were recorded at baseline only. Manufacturer’s cut-off values were used for analyses.

The Sharp-van der Heijde (SvH) scoring method was used to assess radiographic progression of the joints of hands and feet. Anonymised radiographs were read in chronological order [35] by two readers; agreement between them was calculated with intraclass correlation coefficient ICC(A,1). The SvH score consists of the erosion score (0–280) and joint space narrowing (JSN) score (0–168) of hands, wrists, and feet, leading to a maximal score of 448. Progression was defined as a change over time greater than the smallest real difference (SRD), which was derived from the agreement analysis (ICC) between the two readers [36].

### US assessment

**Fig. 1:**
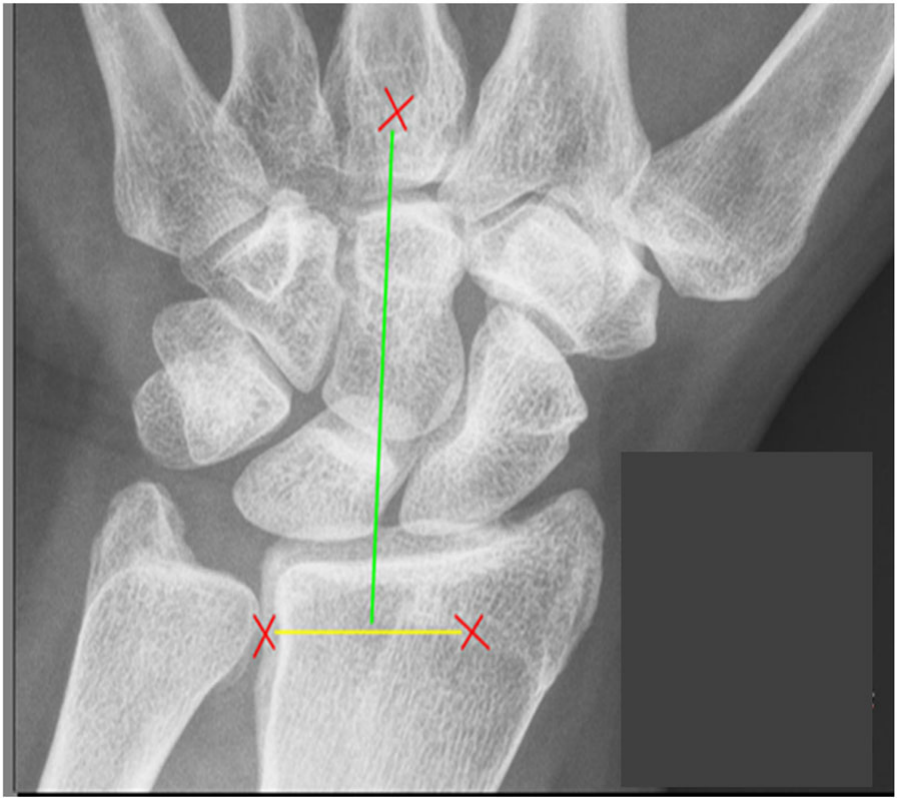
Probe position for scanning the wrist. Bottom two red crosses represent Lister’s tubercle (radial) and the ulnoradial joint (ulnar). The top red cross represents the base of the third digit. Green line shows probe position

For the US examination, each centre used a Esaote MyLab60 machine with a LA-435 probe (linear array 6–18 MHz). The second to fifth metacarpophalangeal (MCP) and proximal interphalangeal (PIP) joints of both hands were scanned in the dorsal and palmar orientation. Bilaterally, radiocarpal, intercarpal, and second to fifth metatarsophalangeal (MTP) joints were scanned dorsally. The ulnocarpal joint was not included because it was found to be the least reliable orientation in which to scan the wrist [37]. Patient and probe positioning were according to EULAR guidelines [38], with the probe position on the wrist specified in more detail (see Fig. 1). Greyscale US (GSUS) and power Doppler US (PDUS) were performed. The GSUS scoring system for synovitis was semiquantitative (0–3), with ‘0’ indicating no US signs of inflammation and ‘3’ indicating severe inflammation, using Naredo’s modification of the scoring system of Szkudlarek and colleague [39], combining the criteria for joint effusion and synovial proliferation. For erosions, the binary scoring system presented by Wakefield and colleagues [40] was modified to a semiquantitative (0–3) scoring system, assigning higher scores to larger erosions. PDUS scores were according to the semiquantitative (0–3) PD scoring system of Szkudlarek and colleagues [39].

For PDUS, the colour gain was set at the disappearance of colour noise, frequency at 10 MHz, a pulse repetition frequency (PRF) of 750 Hz and wall filter (WF) at level 3 out of 5. We adjusted the size and position of the colour box to include the subcutaneous tissue to recognize artefacts caused by vessels above the joint. Based on data in the literature, we considered a joint to be inflamed if it was scored with at least a grade 2 in the GSUS domain and/or at least a grade 1 in the PDUS domain, which resulted in a US joint count (USJC; score 0–28) per patient [41, 42]. For explorative purposes, we investigated different US cut-offs for arthritis in individual joints (e.g. GSUS > 2 and PD > 1) to produce different US sum scores for individual patients.

All seven ultrasonographers, blinded to clinical findings, had been certified for US by the Dutch College of Rheumatology. Prior to the start of the study we organized meetings with all ultrasonographers to discuss the scoring of static images using the scoring systems mentioned above. From these meetings a consensus was reached on how to score images. With this consensus we then composed an atlas with sample images and cartoons for each grade of GSUS, PDUS, and erosive changes. This was distributed to all ultrasonographers, and four training sessions prior to and five more during the study were organized to optimise both interpretation and acquisition reliability between ultrasonographers using this atlas [43].

### Statistical analysis

In our protocol the number of patients with a DAS44 > 3.4 1 year after diagnosis had been chosen as the primary outcome. However, actual data showed that only 10 cases met this criterion. Therefore, we redefined our primary outcome by the absence of DAS28 remission (i.e. DAS28 ≥ 2.6) at 1 year of therapy. Clinical, laboratory, radiographical, US sum scores, and US cut-offs were investigated for association with the absence of DAS28 remission (DAS28 ≥ 2.6) at 1 year using a logistic regression of complete cases. Parameters that were univariately associated with a *p* value < 0.2, or had shown to have prognostic value in the literature, were included in the multivariate model, which was reduced by backward selection (*p* < 0.05). The analyses were repeated after multiple imputation (10 datasets) (results are presented in Additional file 1).

Secondary outcomes were ‘absence of EULAR good response’, analysed with the same analysis as for the primary outcome. Also, ‘presence of radiographical progression’ as assessed by progression on the SvH score greater than the SRD was analysed using a Mann-Whitney *U* test. Additionally, we investigated the association of the extent of inflammation at US at baseline with that at 1-year follow-up. The cross-sectional associations of US arthritis scores and DAS28 were also evaluated at baseline and 1 year using a Spearman correlation coefficient.

In addition, several US sum scores and US cut-offs were tested in multivariate analyses, while keeping the other variables constant. A value of *p* < 0.05 was considered to be statistically significant; all tests were two-sided, and STATA 12, (StataCorp, Texas, USA) was used to analyse the data.

## Results

### Patients

Out of the 174 patients, 159 patients had complete follow-up data (91%); 14 patients dropped out, and for one patient we could not calculate the primary outcome. No significant differences in baseline characteristics were observed between complete (*n* = 159) and incomplete (*n* = 15) cases.

Of the 174 participating patients, 64% were female; the mean (standard deviation (SD)) age was 54.8 (14) years, the median (interquartile range (IQR)) symptom duration was 4.6 (2.8–7.9) months; baseline mean (SD) DAS28 was 4.9 (1.3); 67% of patients were RF positive and 60% were aCCP positive, and 24% of patients had erosions (SvH erosion score > 1) at baseline (Table 1). At 12 months, the mean (SD) DAS28 was 3.5 (0.9) with a median (IQR) of 1 (0–3) swollen joints and 3 (0–5) tender joints; 38% of the patients failed to achieve DAS28 remission and 30% did not achieve the EULAR good response criterion while 3.3% progressed radiographically more than the SRD (6.4 SvH units).

**Table 1:** Baseline characteristics of the patients

Age in years, mean (SD)	54.8 (14)
Symptom duration in months (*n* = 166)	4.6
Gender, % female	64%
DAS28, mean (SD)	4.9 (1.3)
SJC28 at baseline	6 (3–11)
TJC28 at baseline	6 (2–10)
ESR (*n* = 173)	27 (12–47)
VAS, mean (SD)	51 (24)
RF, % positive (*n* = 171)	67%
aCCP, % positive (*n* = 171)	60%
RF and aCCP, % positive (*n* = 171)	54%
HAQ (*n* = 155),	0.88 (0.38–1.25)
USJC	5 (2–9)

*n* = 174, unless specified otherwise

Median (interquartile range) are presented unless specified otherwise

*aCCP* anti-cyclic citrullinated peptide, *DAS* Disease Activity Score, *ESR* erythrocyte sedimentation rate, *HAQ* Health Assessment Questionnaire, *RF* rheumatoid factor, *SD* standard deviation, *SJC* swollen joint count, *TJC* tender joint count, *USJC* ultrasonographic joint count, *VAS* visual analogue scale

### US findings

At baseline (*n* = 174) the median (IQR) joint count per patient regarding synovitis at US (USJC) was 5 (2–9); for erosions at US it was 0 (0–1). The joints most commonly inflamed (GSUS > 1 and/or PDUS > 0) at baseline were radiocarpal joints (right 44%, left 41%), the left MTP2 (36%), and the left MCP2 on the palmar side (35%). Erosions at US were mainly seen in both MTP5 joints (right 23%, left 22%). On x-ray, erosions were seen in 8% and 15% of these respective joints. At 1 year (*n* = 159), median (IQR) USJC score was 0 (0–3) and for US erosions it was 0 (0–1). The most frequently inflamed joints at 1 year were radiocarpal joints (right 14%, left 15%) and the right MCP2 joint (14%). Erosions were found most frequently in MTP5 joints (right 21%, left 25%). On x-ray, erosions were seen in 15% and 21% of these respective joints. Of the 159 patients for whom 1-year US examination was available, 16 patients (10%) did not have inflammation at US at baseline, and 13 (81%) of those also did not have inflammation at US at 1-year follow-up.

We found no cross-sectional association between US inflammation and DAS28 or the individual DAS28 components at baseline or after 1 year. At baseline, the Spearman correlation coefficient for US inflammation score and DAS28 = 0.26, for SJC28 = 0.22, for TJC28 = 0.04, for ESR = 0.24, and for the VAS = 0.12 (all *p* > 0.05). At 1 year, these were similar.

#### Reliability of US data

After four training sessions prior to the start of our study, interobserver reliability (ICC(A,1)) at the joint level between the seven ultrasonographers was 0.58 (95% confidence interval (CI) 0.45–0.72) for GSUS, and 0.48 (0.34–0.64) for PDUS, while it was s 0.58 (0.49–0.68) for the two modalities combined. However, the percentage exact agreement at the patient level (i.e. the agreement of ultrasonographers for classifying a patient as having arthritis or not) was 95%.

### Factors associated with failure of DAS28 remission

Sixty-one patients (38%) failed to achieve DAS28 remission at 12 months. This was associated in the univariate analysis with a higher DAS28 score and higher ESR at baseline (*p* < 0.05). Neither the USJC (GSUS > 1 and/or PDUS > 0) nor US erosion score were associated. Categorizing patients into quartiles based on baseline USJC did not yield significant results, nor did using other definitions of US inflammation. Building a multivariate model resulted in a final model with baseline DAS28, RF, age, and the monitoring strategy with the shortest assessment interval (i.e. every 4 weeks) (Table 2). Adding US results to the final model did not significantly improve the fit of the model (LR Chi^2^ = 1.04; *p* = 0.31). The analysis with imputed data yielded almost equal results (see Additional file 1).

**Table 2:** Univariate and multivariate association of relevant patient characteristics with the outcome: absence of DAS28 remission at 12 months

Variable	Univariate		Multivariate	
	OR (95% CI)	*P* value	OR (95% CI)	*P* value
DAS28 per point	1.34 (1.03–1.73)	0.03	1.60 (1.18–2.2)	0.002
Female gender	1.94 (0.96–3.3.9)	0.06	1.93 (0.91–4)	0.09
RF positive	1.78 (0.87–3.63)	0.11	2.34 (1.07–5.1)	0.03
aCCP positive	1.54 (0.79–3)	0.21		
RF and aCCP positive	1.59 (0.83–3)	0.17		
Age per year	1.01 (0.99–1.04)	0.24		
Monitoring strategy				
Sub-cohort A	Reference	NA	Reference	NA
Sub-cohort B	0.49 (0.13–1.73)	0.27	0.20 (0.05–0.85)	0.03
Sub-cohort C	1.26 (0.47–3.39)	0.64	0.68 (0.20–2.4)	0.55
Sub-cohort D	1.26 (0.26–1.48)	0.28	0.85 (0.30–2.4)	0.76
Symptom duration (months)	1.0 (0.91–1.1)	0.99		
SvH per point	1.04 (0.93–1.15)	0.48		
Smoking yes/no	1.56 (0.73–3.4)	0.25		
HAQ per point	1.16 (0.71–1.9)	0.55		
USJC per point	0.99 (0.92–1.06)	0.77	0.96 (0.89–1.04)	0.31

Absence of remission = 1

Ultrasound joint count (USJC) = number of joints with at least greyscale (GS) > 1 or power Doppler (PD) > 0

Multivariate model including: DAS28 at baseline, RF, monitor strategy, gender, and USJC

Monitor strategies: Sub-cohort A, monitoring at least every 3 months and treatment escalation at the discretion of the physician; sub-cohort B, monitoring every 4 weeks and treatment escalation if no remission; sub-cohort C, monitoring varies between 4 and 12 weeks and treatment escalation if no remission, prespecified in a protocol [24]; sub-cohort D, monitoring every 3 months and treatment escalation if no low-disease activity [23]

*aCCP* anti-cyclic citrullinated peptide, *CI* confidence interval, *DAS* Disease Activity Score, *HAQ* Health Assessment Questionnaire, *NA* not applicable, *OR* odds ratio, *RF* rheumatoid factor, *SvH* Sharp-van der Heijde

### Effect of different US sum scores and cut-offs on the multivariate analysis

In our primary analysis, joints were considered to be inflamed at US if GSUS > 1 and/or PDUS > 0. We also evaluated several US sum scores and US cut-offs. No US sum score or cut-off was univariately significantly associated with the outcome. The coefficients of the US parameters did not significantly change either when testing all US sum scores and US cut-offs in a multivariate analysis (Table 3).

**Table 3:** Results of logistic regression analyses with different US definitions predicting the absence of DAS28 remission at 1 year

Ultrasound score	OR (95% CI)
Per point of:	
GS	0.99 (0.96–1.02)
PD	0.99 (0.95–1.03)
Number of joints with:	
GS > 1	0.67 (0.27–1.71)
PD > 0	0.80 (0.30–2.16)
GS > 1 and/or PD > 0	0.96 (0.89–1.04)
GS > 1 or PD > 0	0.96 (0.42–2.23)
GS > 2	0.56 (0.27–1.20)
GS > 2 or PD > 0	0.57 (0.27–1.21)
GS > 0 and PD > 1	0.82 (0.39–1.7)
GS > 0 and PD > 2	1.12 (0.41–3.01)
GS > 1 and PD > 2	1.12 (0.41–3.01)
GS > 2 and PD > 1	0.71 (0.32–1.56)
GS > 2 and PD > 2	1.26 (0.37–4.24)
GS > 0 or PD > 0	0.40 (0.12–1.33)

Continuous measures: ultrasound joint count = number of joints with greyscale (GS) > 1 and/or power Doppler (PD) > 0; GS sum = sum of GS scores; PD sum = sum of PD scores; GS > *x*, presence of a joint(s) with a GS > *x*; PD > *y*, presence of a joint with a PD > *y*

*CI* confidence interval, *OR* odds ratio

### Secondary outcomes

We evaluated the prediction of not achieving the EULAR good response criterion, radiological progression, and presence of inflammation at US at 12 months as secondary outcomes; 30% of patients did not achieve EULAR good response. Adding US results to the final model did not significantly improve the fit of the model (LR Chi^2^ = 0.98; *p* = 0.32). Only five patients showed radiographical progression larger than the SRD. These patients numerically had a higher grade of US joint inflammation at baseline compared to those without radiographical progression: median (IQR) USJC 8 (7–10) versus USJC 5 (2–9), respectively; this difference did not reach statistical significance (*p* > 0.05). Those with a USJC of 0 at 12 months had a median (IQR) USJC of 4 (1–7) at baseline, whereas those with USJC > 0 at follow-up had a median (IQR) USJC of 6 (4–10) at baseline (*p* = 0.003).

## Discussion

Results of routinely performed US at baseline show no additional prognostic information when added to clinical, laboratory, and radiographical parameters in predicting failure to achieve DAS28 remission at 1 year in early RA patients treated to target. The absence of remission was associated with high DAS28 at the time of diagnosis and RF positivity, confirming data in the literature [12–14]. The monitoring strategy with the shortest assessment interval (4-week visits), corrected for DAS28 and RF positivity at baseline, appeared to increase the chance to achieve remission.

The absence of a predictive value of US in this study contrasts with data showing that US predicts patients with undifferentiated arthritis (UA) progressing to RA [16, 17] and RA patients in remission progressing to flare and erosion [18–21]. Our negative study results may be explained by the earlier observation that intensive therapy diminishes the prognostic value of baseline conventional prognostic factors [8]. Our negative results are in line with earlier observations in a smaller population of RA patients not treated to target, 64% of whom received glucocorticoids prior to inclusion in the study [26]. However, in that study ‘time integrated values’ of PDUS correlated with clinical outcomes, which was not found in our study.

It has been suggested that one of the most important steps we have yet to take in US research in RA is to investigate the added value of US and clinical indices in multicentre trials for clinical and radiological outcomes of RA patients [44]. Our study has done this. Although our study has shown that the added predictive value of routinely performed US at baseline in early RA patients is absent if treated to target, US still has a place in personalised medicine for specific indications. For example, in patients for whom the widely used DAS28 is less valid, e.g. patients with concomitant fibromyalgia who will have a high tender joint count and high VAS, and also for obese patients in whom the clinical joint examination may be difficult and who may have a higher ESR without joint inflammation [45]. In these patients, US may really make a clinically relevant contribution. US might help to distinguish patients with or without joint inflammation making more personalised medicine possible. To that aim, new trials should be performed using US measures as target for treatment, or using US as a basis for treatment escalation and tapering; initiatives using US as an outcome measure have been published [46, 47]. In our opinion, the observation that more than 80% of the patients who did not have US inflammation at baseline also did not have US inflammation at follow-up deserves further investigation.

A limitation of our study was heterogeneity in the strategies and assessment intervals of the sub-cohorts from which we included patients. All patients, however, were treated according to a ‘treat to target’ and ‘tight control’ strategy’. Our patients had relative mild disease with a median six swollen joints at baseline; however, this reflects an early RA population and application of the more sensitive, newest RA classification criteria [24]. Both are aspects of normal daily clinical practice in which future US trials as suggested above should also be performed [44]. Another limitation may be the use of US equipment that now might be seen as midrange and less sensitive compared to current high-end machines. However, this also reflects routine practice. More importantly, however, the PD modality in the machines used is actually very sensitive, even better than that of professional research machines [48]. Another possible limitation of our study is that we did not scan all joints included in the DAS28; it could be argued that we missed US inflammation. However, more than 90% of the patients in our study had US inflammation at baseline, and US scoring of a limited sets of joints also well reflects RA activity [49, 50]. A last issue of critical discussion is the moderate agreement coefficients between ultrasonographers, despite organizing several trainings sessions to increase reliability. Generally, reliability in ultrasound is not a bigger problem than in other imaging modalities if performed by trained examiners [51], as was the case in our study. That the agreement coefficients are moderate is probably largely attributable to relatively low levels of detectable inflammation at US in the patients participating in the trainings sessions; ICC values are low in samples with a homogeneous distribution, i.e. with only very high or very low scores of arthritis. Since the heterogeneity of the scores in the patient population largely determines the value of reliability measures [43], we tried to select joints from the patients for the training sessions with scores ranging from 0 to 3. Apparently, we did not succeed in doing this well enough. The hypothesis that the reliability coefficients in our study underestimate reliability is corroborated by the observation that the percentage exact agreement at the patient level was 95%.

## Conclusion

In conclusion, in an early RA population, treated according to the ‘treat to target’ paradigm, adding data from US of the joints at baseline to the work-up did not improve prediction for not achieving DAS28 remission or EULAR good response, nor the prediction of radiographical progression at 1 year.

## Additional file

Additional file 1: Description of data: univariate and multivariate logistic regression in the multiple imputation dataset (M = 10) using STATA 12 and REALCOM. (DOC 44 kb)

## Abbreviations

aCCP: Anti-cyclic citrullinated peptide
DAS: Disease Activity Score
DHAQ: Dutch Health Assessment Questionnaire
DMARD: Disease-modifying anti-rheumatic drug
ESR: Erythrocyte sedimentation rate
GSUS: Greyscale ultrasound
ICC: Intraclass correlation coefficient
IQR: Interquartile range
MCP: Metacarpophalangeal
MTP: Metatarsophalangeal
PDUS: Power Doppler ultrasound
RA: Rheumatoid arthritis
RF: Rheumatoid factor
ROMA: Reumatologie Online Monitoring Applicatie
SD: Standard deviation
SJC: Swollen joint count
SRD: Smallest real difference
SvH: Sharp-van der Heijde
TJC: Tender joint count
tREACH: Treatment in the Rotterdam Early Arthritis Cohort
US: Ultrasonography
USJC: Ultrasound joint count
VAS: Visual analogue scale
