# Supplementary material for: Can baseline ultrasound results help to predict failure to achieve DAS28 remission after 1 year of tight control treatment in early RA patients?
Source: Arthritis Res Ther. 2018 Jan 30;20:15. doi: 10.1186/s13075-018-1514-2 (PMC5791342; doi:10.1186/s13075-018-1514-2)
Supplement: Additional file 1: — Description of data: univariate and multivariate logistic regression in the multiple imputation dataset (M = 10) using STATA 12 and REALCOM. (DOC 44 kb) [file 13075_2018_1514_MOESM1_ESM.doc]

Patient flow

174 out of 194 initial selected RA patients were available for analysis. As treatment allocation was concealed in two cohorts, 11 out of the initial 194 patients were excluded post hoc because they had not received at least a potent DMARD at start of therapy. Another 9 patients were excluded: 5 received treatment before the US examination, 2 were diagnosed later with an inflammatory joint disease other than RA, 1 did not show up for any visit and in 1 incorrect PDUS settings had been used.

Out of the 174 patients, 159 patients did have complete follow up data (91%). 14 patients dropped out, among whom one patient who died and one patient who was diagnosed with terminal cancer, both unrelated to this study. And for one patient we could not calculated the primary outcome. No significant differences in baseline characteristics were observed between complete (n=159) and incomplete cases (n=15).

12 Month results were presented for the complete cases (n=159). These analysis were repeated for the 174 patients that initial were available for analysis. The results are shown below.

Additional file 1: Univariate and multivariate logistic regression in the multiple imputation dataset (M=10) using STATA 12 and REALCOM

| **Variable** | **Univariate** |  | **Multivariate** |  |
| --- | --- | --- | --- | --- |
|  | **OR (95% CI)** | **p-value** | **OR (95% CI)** | **p-value** |
| DAS28 per point | 1.31 (1.00 – 1.69) | 0.04 | 1.53 (1.12 – 2.08) | 0.007 |
| Female gender | 1.84 (0.91-3.72) | 0.09 | 1.94 (0.76-2.86) | 0.09 |
| RF positivity | 1.72 (0.82 – 3.58) | 0.15 | 2.23 (0.97-5.12) | 0.06 |
| aCCP positivity | 1.62(0.84-3.13) | 0.15 |  |  |
| RF and aCCP positivity | 1.65(0.87-3.14) | 0.13 |  |  |
| Age per year | 1.01(0.99-1.04) | 0.28 |  |  |
| Monitor strategy  A  B  C  D | A. 1.00 (-)  B. 0.53 (0.15-1.85)  C. 0.96(0.31-3)  D. 1.29 (0.43-3.22) | A. NA  B. 0.27  C. 0.95  D. 0.63 | A. 1.00 (-)  B. 0.24 (0.06-0.98)  C. 0.66 (0.19 – 2.22)  D. 0.96 (0.31-2.98) | A.NA  B. 0.05  C. 0.5  D. 0.94 |
| Symptom duration (mo.) | 1.0 (0.94 – 1.1) | 0.85 |  |  |
| SvH units | 1.04 (0.93 – 1.16) | 0.47 |  |  |
| Smoking | 1.56 (0.73 – 3.4) | 0.25 |  |  |
| Alternative HAQ | 1.16 (0.71-1.9) | 0.55 |  |  |
| USJC per point | 0.98 (0.92 – 1.05) | 0.68 | 0.96 (0.89 – 1.04) | 0.31 |
